# Prediction of Hole Expansion Ratio for Advanced High-Strength Steel with Image Feature Analysis of Sheared Edge

**DOI:** 10.3390/ma16072847

**Published:** 2023-04-03

**Authors:** Kyucheol Jeong, Yuhyeong Jeong, Jaewook Lee, Chanhee Won, Jonghun Yoon

**Affiliations:** 1 Department of Mechanical Design Engineering, Hanyang University, Seoul 04763, Republic of Korea; kcjeong0@gmail.com (K.J.); jtpye9402@gmail.com (Y.J.); 2 Department of Mechanical Engineering, BK21 FOUR ERICA-ACE Center, Hanyang University, Ansan 15588, Republic of Korea; 3 Materials Forming Research Group, POSCO Global R&D Center, Incheon 21985, Republic of Korea; leejaewook@posco.com; 4 Digital Transformation R&D Department, Korea Institute of Industrial Technology, Ansan 15588, Republic of Korea; chan2@kitech.re.kr

**Keywords:** advanced high-strength steels (AHSS), shear-affected zone, edge cracking, sheared edge, hole expansion ratio (HER), machine vision

## Abstract

The punching process of AHSS induces edge cracks in successive process, limiting the application of AHSS for vehicle bodies. Controlling and predicting edge quality is substantially difficult due to the large variation in edge quality, die wear induced by high strength, and the complex effect of phase distribution. To overcome this challenge, a quality prediction model that considers the variation of the entire edge should be developed. In this study, the image of the entire edge was analyzed to provide a comprehensive evaluation of its quality. Statistical features were extracted from the edge images to represent the edge quality of DP780, DP980, and MART1500 steels. Combined with punching monitoring signals, a prediction model for hole expansion ratio was developed under punch conditions of varying clearance, punch angle, and punch edge radius. It was found that the features of grayscale variation are affected by the punching conditions and are related to the double burnish and uneven burr, which degrades the edge quality. Prediction of HER was possible based on only edge image and monitoring signals, with the same performance as the prediction based solely on punching parameters and material properties. The prediction performance improved when using all the features.

## 1. Introduction

Regulations pertaining to the energy efficiency of vehicles have significantly impacted the choice of body-in-white (BIW) materials. There has been an increase in the use of GPa-grade steels or advanced high-strength steels (AHSS) because of their high strength-to-weight ratio [1], which contributes to a high energy efficiency for transportation.

Typically, the process of creating BIWs involves flanging, which consist of stretching the edge fabricated by the mechanical blanking. If the edge of AHSS is stretched, a crack is observed at the early stage of the flanging process compared with a conventional steel sheet with medium strength, which implies that AHSS is sensitive to the damage induced by the mechanical shearing process. Branagan et al. [2] compared the stretchability of edges fabricated by punching, electrical discharge machining (EDM), milling, and laser cutting. They reported that for the third generation AHSS, the hole expansion ratio (HER) of punching was 6–12%, whereas the other methods had an HER value of 65–140%. In addition, AHSS has a high variance in edge-failure strain [3], which makes the design of punching and flanging processes challenging.

Typical AHSS consists of multiple phases. Dual-phase (DP) steel, which has a relatively high ductility, consists of ferrite and second-phase martensite but has limited strength. In contrast, Martensite (MART) steel has a martensite matrix with small amounts of bainite and ferrite, showing high strength but limited ductility [4,5]. The phase distribution and its microstructure affect the fracture mechanism, which results in the low edge stretchability and high-variance edge stretchability of AHSS [6,7,8].

The crack during the flanging process relies on the characteristics of the edge where classical FLD is not able to predict failure [9,10]. Hence, the test for failure criteria for the flanging process is directly designed as flanging and is standardized according to ISO-16630 [11], namely, the hole expansion test (HET) and hole expansion ratio (HER). In this test, a conical punch is used to expand a hole in a specimen until it fractures. As the test is destructive, the resulting HER value is stochastic, i.e., estimates the average and variance of the quality of the edge. However, due to the large variations in AHSS, it does not guarantee the edge quality of each individual product. Furthermore, the high strength of the AHSS can induce rapid wear and lead to changes in punching conditions, thereby limiting the HER estimation based solely on punching parameters [12]. Therefore, there is a need for a non-destructive, rapid, and inexpensive method to measure the edge quality which enables the inspection of each individual edge with large quality variation.

Many methods for edge damage quantification have been suggested to understand the underlying physical phenomena or to provide an estimation of edge damage, such as microhardness [13,14,15], metal flow angle [16], void inspection [17,18], and geometry of the edge. Except for the edge geometry, the methods require microscopy inspection and cutting of the specimen, limiting its application.

**Figure 1:**
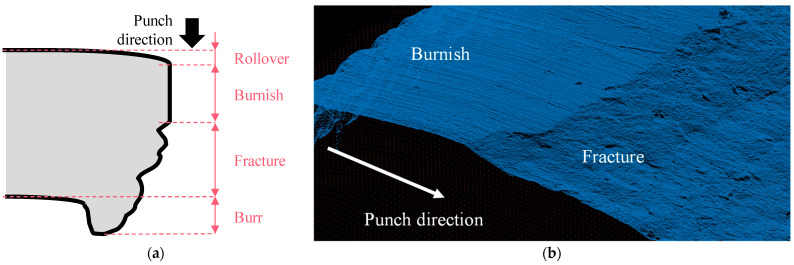
Classification of edge geometry in (**a**) schematic illustration and (**b**) 3D microscopy.

An illustrative diagram of the edge geometry is shown in Figure 1a, and 3D microscopy data of the surface is given in Figure 1b. The conventional geometrical characteristics include the lengths of the rollover, burnish, fracture zone, and burr. The rollover zone is related to the material flow induced by blanking [19], and its length is affected by the material properties [20]. The burnish zone is the smooth impression induced by the punch penetrating the metal sheet, whereas the fracture zone is a rough surface caused by a crack [21]. The burr is generated at the end of the fracture zone. It is reported that the punching parameter, geometrical characteristics, and edge quality are correlated each other for DP steels [16]. Zhou et al. [22] reported that the high-tensile performance of QP980 was observed when the burnish and burr lengths were short. In addition, the undulated burrs decreased the tensile performance. Mori et al. [23,24] observed that when the clearance is as low as 4%, a secondary burnish zone is formed on JSC980Y—the maximum hardness observed at the boundary of secondary burnish. According to these studies, the punching parameter that affects edge stretchability has a significant effect on the geometrical characteristic length. Based on these trends, Wang et al. [25] collected data on four characteristic lengths and edge stretchability under various trimming conditions using an aluminum 6000 series.

The geometry of edges can be measured with 3D microscopy [25], replica techniques with image processing [16], and panoramic images of cut edges [26]. Although these measurement methods have already been suggested, they are still used to measure only the characteristic zone length or to perform qualitative analyses. To the best of our knowledge, no damage quantities have been defined for the entire sheared-edge surface with the non-destructive measurement method.

In addition to measuring the edge, quality can be estimated through process monitoring. Recently, several studies have been conducted on blanking monitoring. Many studies on process diagnosis have been conducted using time series such as acoustic emission (AE) and punch loads. Studies on blanking process monitoring have focused on detecting the effects of wear on the signals. Unterberg et al. [27] analyzed the relationship between the number of punch stroke cycles and the temporal, statistical, and spectral features of AE. Hoppe et al. [28] analyzed the relation between clearance, tool radius, and load data. Kubik et al. [29] evaluated the effect of tool edge radii on the load and strain data. Niemietz et al. [30] proposed a wear-prediction model that quantified the fine-blanking punch wear, based on the load data. The aforementioned above [27,28,29,30] used medium-strength steel, and the sensor was well-integrated into a blanking machine. In this study, the range of the ultimate tensile strength of the steels was 780–1500 MPa. The details of the signals and features are presented in Section 3.1.

**Figure 2:**
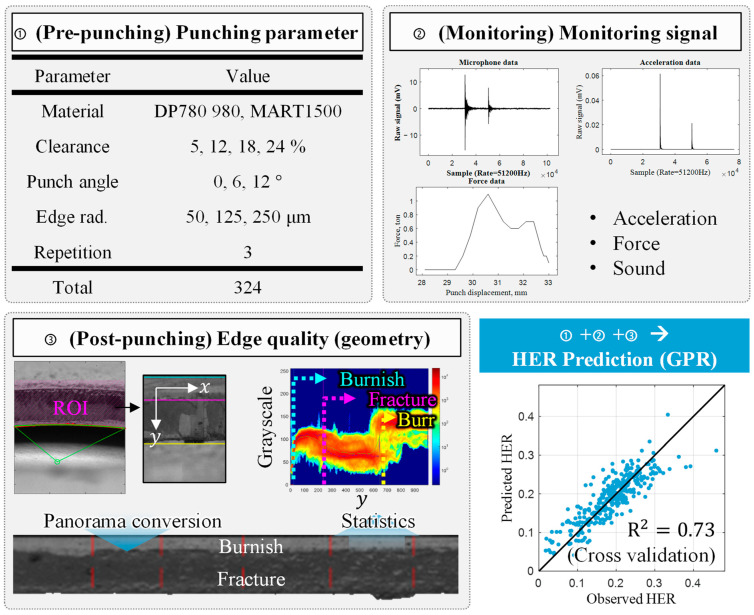
Overall procedure for predicting the HER value.

The purpose of this study is to propose a prediction model for edge stretchability that reflects the variation in quality along the entire edge and can be easily and rapidly measured. A prediction model was proposed using features extracted from monitoring the data and geometry of the entire edge as shown in a graphical abstract in Figure 2. Three AHSS materials with a large range of strength, DP780, DP980, and MART1500, were punched under various conditions, as described in Section 2. Section 3 explains how the sound, vibration, and load monitoring data are measured and how the features are extracted. The details of the image processing and features are provided, which were newly suggested in this study. Section 4 provides a detailed explanation of the HER prediction model using a dataset under various punching conditions. The Gaussian process regression model, HER prediction performance, and analysis of the optimized features are explained.

## 2. Materials and Experiments

The experiments were designed to investigate the relationship between the data of punching process characteristics and the HER value. To achieve this, different AHSS materials with varying strengths and punching conditions were prepared, and various types of data were collected. The data used to evaluate edge quality were categorized as pre-punching, punching monitoring, and post-punching data. Pre-punching data include experimental set-up for punching, such as punching process parameters and material properties. Punching monitoring data comprised real-time diagnosis of the process, including sound, vibration, and punch loads. Post-punching data refer to measurement data collected after the punching process was complete. The image of the sheared edge was used due to its rapid, non-destructive, and macroscopic properties of the capturing process. All data and features of the prediction model are described in Table 1, and their experimental methods are explained in this section. Further explanation regarding the types and extraction of features is provided in Section 3.

### 2.1. Experimental Set-Up for Punching Process

**Figure 3:**
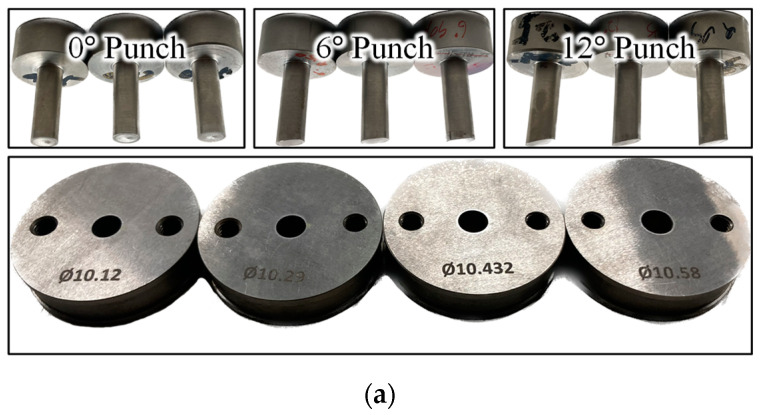

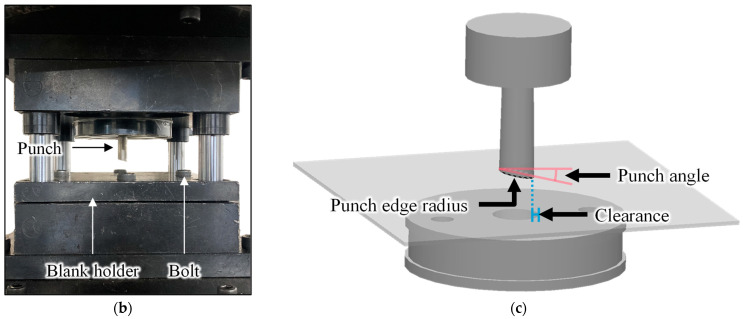
Experimental set-up for punching process: (**a**) die set, (**b**) punching set-up for the experiment, and (**c**) process parameters in punch and die.

To ensure a wide range of AHSS strengths, three materials (DP780, DP980, and MART1500) commonly used for vehicle parts but known to have edge cracking were provided by POSCO. The selected AHSS materials varied in strength from 780 MPa to 1500 MPa, and their mechanical properties are listed in Table 2—along rolling direction (RD), transverse direction (TD), and diagonal direction of 45°. The tensile test had been conducted with DIC and the results were given for true stress–strain. All sheets used in the experiments had a thickness of 1.2 mm. Punching was conducted using a 100-ton press machine (Figure 3b). Punch speed was set as 5 mm/s since the speed range of 1~25 mm/s had no effect on the HER of DP780 and DP980 [31].

To investigate and fabricate the sheared edge, the type of specimen used for punching should be considered. For this study, the shape of the specimen edge was fixed to a hole geometry for HET. Although a linear-shaped edge [32,33] is more advantageous for visual inspection, the anisotropic geometry of the specimen can induce stress concentration effects that affect crack initiation. In this case, the geometry of the edge is less relevant to the edge quality.

The punching process parameters related to both edge quality and wear were selected. The design parameters are shown in Figure 3c. Clearance is the gap between the punch and die, which is defined as a percentage of the sheet thickness. For AHSS, the best edge qualities usually lie in the clearance range of 8–15%. In this experiment, the range of clearance was set as 5–24% to investigate various edge qualities. The other parameters were punch angle and punch-edge radius, which are the inclined angle and the fillet radius at the tip of the punch, respectively. The punch angle had been reported to significantly affect both HER and edge geometry in DP steel [34]. During AHSS trimming, the punch-edge radius and clearance increased due to the effect of abrasive wear [12], which led to a decrease in edge stretchability. Long burrs [35] and low edge stretchability [25] were observed when the tool edge radius was large. The ranges of punch angles and punch-edge radii selected in this study were set to reflect these change in edge quality and are listed in Table 3. The punch and die sets used in this study are shown in Figure 3a. Carburized SKD11 were used for punch and die. The punch diameters were fixed to 10mm according to HER standard ISO-16630 [11].

### 2.2. Experimental Set-Up for Punching Monitoring

**Figure 4:**
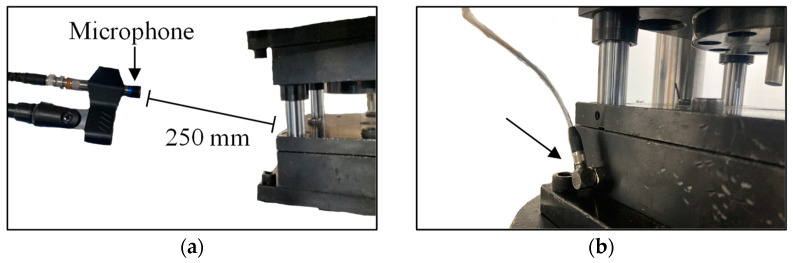
Experimental set-up for punching monitoring: position of (**a**) microphone and (**b**) accelerometer.

In order to investigate the effect of the process monitoring, signals on HER, sound, die vibration, and punch load were recorded. The punch load was measured using a load cell, while sensors that were easy to implement on any die were used to measure sound and vibration. An external microphone (GRAS 146AE) and an accelerometer (Kistler 8763B050BB) were placed and attached to the die. The microphone and accelerometer are shown in Figure 4a,b, respectively. Monitoring methods were fixed and remained unchanged during the entire experiment.

### 2.3. Experimental Set-Up for Edge-Image Capture

**Figure 5:**
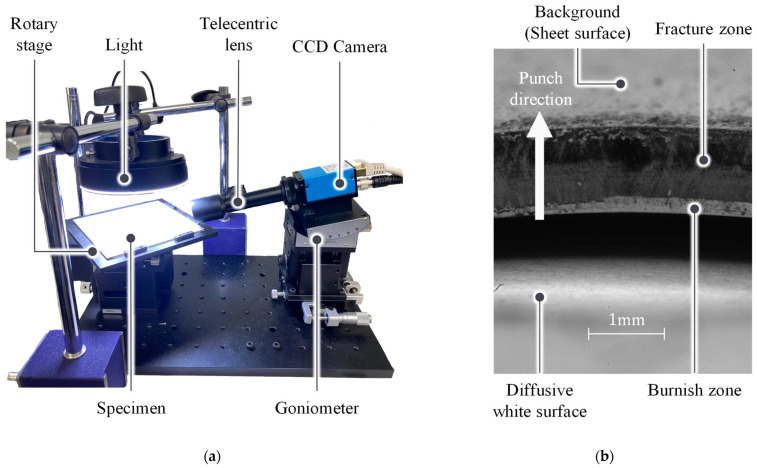
Experimental set-up for edge capturing: (**a**) camera setup for capturing the edge, and (**b**) example of the captured edge.

A set-up for image capture was designed to provide rapid and non-destructive inspection along the entire edge. Figure 5a shows the set-up for image capture. A charge-coupled device (CCD) camera with a 5 MP resolution (DMK 33GP031) was used to capture the images. A Telecentric lens with a magnification of 1x and depth of field (DOF) of 1.5mm was selected. The lens selection was limited by the DOF because of the depth variation in the edge geometry. In addition, the hole shape requires the camera to be inclined, which requires additional DOF. Hence, the trade-off between the zoom magnification and DOF limits the zoom to 1×.

The camera was inclined using a goniometer to capture the hole edge. A rotary stage was used to capture the entire 360° edge. A ring light was used to provide light that was unaffected by rotation. Figure 5b shows an example of an edge image in which the rollover is downward, and the burr is upward. The floor of the rotary stage is a diffusive white surface; therefore, the scatter-reflected light makes the burnish bright and the fracture dark. The gain and exposure of camera were fixed to constant.

The pixel unit can be transformed into a length unit based on the angle of camera tilting. If distortion of telecentric lens is ignored, the following ratio can be derived from the geometric relationship of the specimen, camera, and lens

(1) $$\frac{1pixel}{1\mu m}=\left(l_{ccd}\times \frac{1}{M}\right)\left(\frac{1}{\cos\theta_{c}}\right)=\left(2.2\times 1\right)\left(\frac{1}{\cos13.2{}^{\circ}}\right)\approx 2.26$$

where $l_{ccd}$ is the size of the CCD in μm/pixel, M is the magnification of the lens, and $\theta_{c}$ is the tilting angle of the camera.

**Figure 6:**
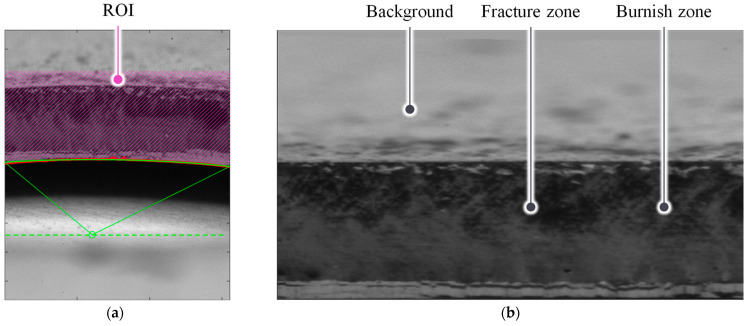
Image coordinate conversion from (**a**) cylindrical raw image to (**b**) Cartesian image.

To extract the region of interest (ROI), the boundary between rollover and burnish was extracted with global threshold and blob analyses. To transform a cylindrical ROI (Figure 6a) into Cartesian image (Figure 6b), the ellipse was fitted to the extracted boundary, where the lengths of major axis and minor axis were set as constant calculated from the camera set-up. Subsequently, the transformation between cylinder coordinates and Cartesian coordinates was performed using the parameter of fitted ellipse. Images were captured at 360° and intervals of 10°. All 36 images were combined into one panoramic image using KAZE feature [36] matching.

### 2.4. Hole Expansion Test (HET)

**Figure 7:**
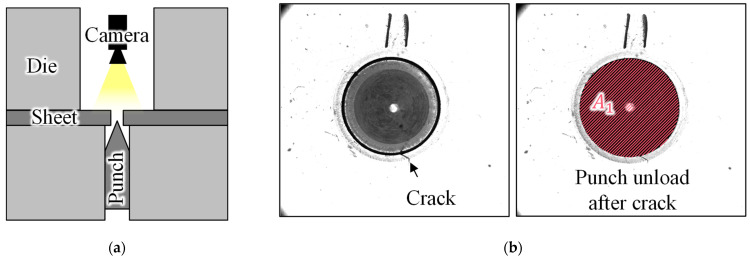
HET process: (**a**) schematic of HET and (**b**) area measurement after crack occurrence.

The HER value was used as quantity for the edge damage. All HET in this study is conducted with the corresponding standard ISO-16630 [11]. When the crack was detected, using a camera as shown in Figure 7, the punch was unloaded, and the HER was calculated based on the initial hole area $A_{0}$ and expanded hole area $A_{1}$ as

(2) $$HER=\frac{d_{1}-d_{0}}{d_{0}}=\frac{\sqrt{A_{1}/\pi }-\sqrt{A_{0}/\pi }}{\sqrt{A_{0}/\pi }}$$

The initial pixel area $A_{0}$ is calculated based on camera and telecentric lens specification.

## 3. Data Collection and Feature Extraction in Sheared Edge

The collected data consist of various types, such as time series and images. The widely used feature types for monitoring are temporal, statistical, and spectral. However, the punching of AHSS was rapid which limits the usage of the features, as described in Section 3.1. For images, there are no such general libraries for features or analysis methods. Hence, the statistical features were suggested in Section 3.2 to reflect the variation along the entire edge, which is related to the edge quality.

### 3.1. Punching Monitoring

**Figure 8:**
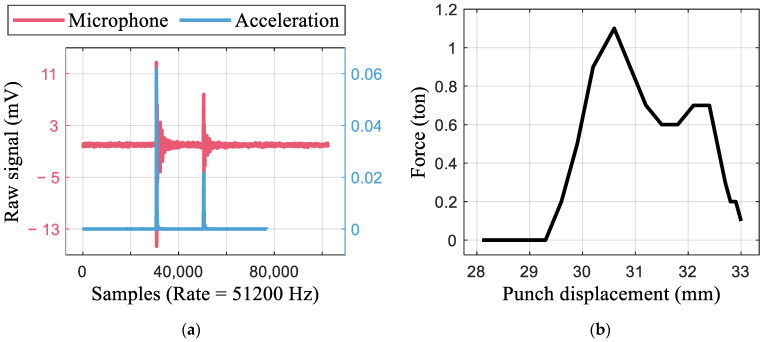
Example signal of (**a**) microphone and accelerometer and (**b**) force punched by punch with angle.

Figure 8a,b show examples of the monitoring signals. Although a relatively low punch speed of 5 mm/s was used, the sound and acceleration signals were pulses. Additionally, the spectral characteristics of sound and vibration were affected not only by the punching parameters, but also by the natural frequency of the die. Hence, depending on the characteristics of the signal, only the intensity of the signal was considered. For the time series of the punching phase $\vec{a}$, the extracted features are the maximum signal intensity $\max\left|\vec{a}\right|$, and the signal energy, defined as

(3) $$E_{signal}=\sum \left|a_{t}\right|$$

The punching phase of a sound and vibration signal can be distinguished by using a threshold. For a load time series, the punching phase was defined based on the derivative of the load signal.

### 3.2. Post-Punching Measurement (Edge Image)

**Figure 9:**
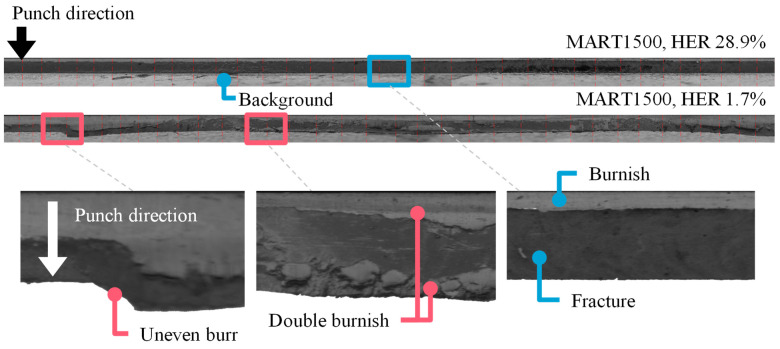
Panoramic images of the highest and lowest HER specimens for MART1500.

Panoramic images of the specimens with the highest and lowest HER for MART1500 are shown in Figure 9. In Figure 9, the highest HER specimens had (1) a short burnish zone length and a long fracture zone length, (2) a fracture zone with a homogeneous grayscale distribution, and (3) a short burr. In contrast, the specimens with the lowest HER exhibited (1) a long burnish zone, (2) burnish zone in fracture zone, or double burnish, and (3) long, uneven burrs. It had been reported [23,24] that double burnish was typically created when the clearance is extremely low as 4%, and an abrupt geometry change at the double burnish degrades the edge quality. However, in this experiment with AHSS materials, double burnish was formed at a large clearance of 24% with a punch angle of 6°.

**Figure 10:**
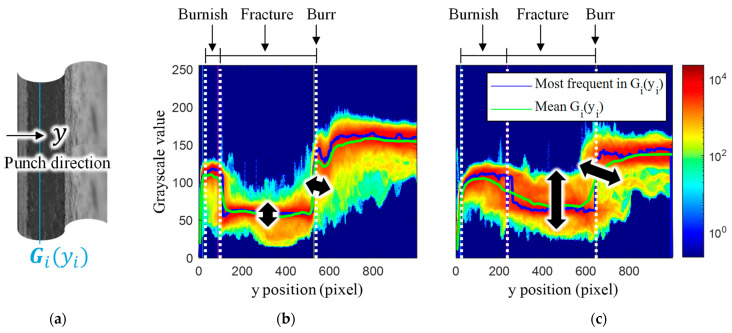
Bivariate histogram of the panoramic image: (**a**) extraction of grayscale histogram; (**b**,**c**) comparison of bivariate histogram between (**b**) high-HER and (**c**) low-HER specimen.

The features of the panoramic images were selected to reflect these phenomena, such as double burnish and uneven burr. First, the conventional characteristic zone was defined to divide the section of the panoramic image. Grayscale statistics were used to calculate the characteristics lengths. A grayscale column was extracted at each $y_{i}$ position, $G_{i}(y_{i})$, as shown in Figure 10a. For all i, the $G_{i}(y_{i})$ is represented by a bivariate histogram. Figure 10b,c are the bivariate histogram of the highest HER specimen and the lowest HER specimen of MART1500, the same specimen shown in Figure 9. Colors indicated counts on a logarithmic scale. At a low y, the grayscale value was relatively high because the burnish zone accounts for most of the edge geometry. If y increases, a portion of the fracture zone begins to dominate the edge geometry, as indicated by a decrease in grayscale. After the burr line, the grayscale increased owing to the bright background. In Figure 10b,c, the blue and green lines represent the most frequent and mean grayscale values for $G_{i}(y_{i})$, respectively. The most frequent grayscale value exhibited a rapid change at the grayscale level, where the mean grayscale value changed smoothly. Hence, the characteristic length and burr location are defined based on the most frequent grayscale values. Figure 10b,c depicts the start of burnish, fracture zone, and burr. Edge geometry characteristics were observed in the bivariate histogram. In the fracture zone, a large grayscale variation was observed for the low-HER specimens, whereas the high-HER specimens exhibited a relatively small variation. The low-HER specimen has a long burr length and large grayscale variation in the y direction, which means that the burr formation is uneven, whereas the high-HER specimen has a short burr and uniform burr line.

**Figure 11:**
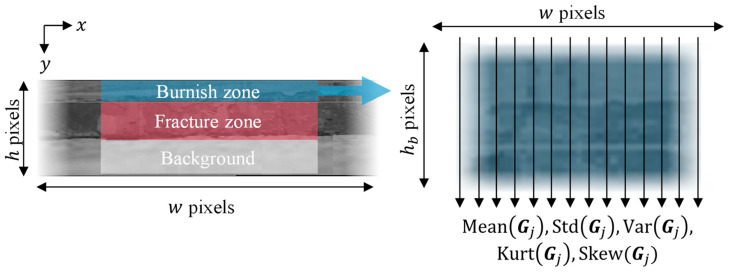
Extraction of statistical features of panoramic images.

After defining the characteristic zones, the features of the panoramic images were extracted for each zone. As shown in Figure 11, the mean, standard deviation, variance, kurtosis, and skewness of the grayscale were calculated for each column. If the width of the burnish zone image is w pixels and grayscale row is denoted as $G_{j}(x_{j})$, $Mean\left(G_{j}(x_{j})\right),Std\left(G_{j}(x_{j})\right),\ldots$ are vectors with length w. Then, the statistical feature matrix of the burnish zone is defined as

(4) $$S_{b}=[Mean\left(G_{j}\right),Std\left(G_{j}\right),Var\left(G_{j}\right),Kurt\left(G_{j}\right),Skew\left(G_{j}\right)]$$

which is w×5 matrix where w is width of the panorama image. Subsequently, the statistical features of $S_{b}$ were calculated to yield a feature vector of length 25.

(5) $$f_{b}=[Mean\left(S_{b}\right),Std\left(S_{b}\right),\;Var\left(S_{b}\right),Kurt\left(S_{b}\right),Skew\left(S_{b}\right)]$$

The same process was repeated for the fracture zone.

**Figure 12:**
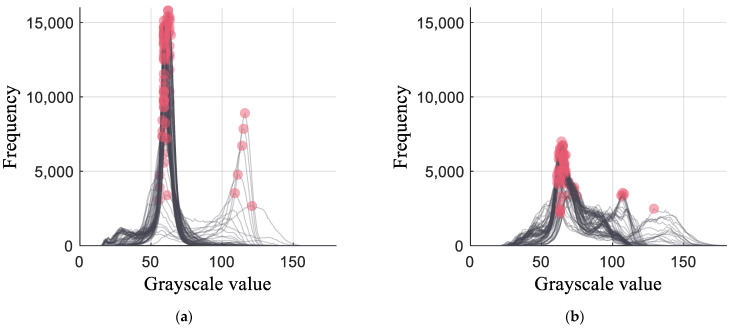
Grayscale histogram at the fracture zone of (**a**) MART1500 specimen with the highest HER and (**b**) MART1500 specimen with the lowest HER.

To effectively reflect double burnish phenomena, the peak frequencies were extracted for each grayscale histogram $G_{i}(y_{i})$. Figure 12a,b depict the top two extracted peaks in the fracture zone of the MART1500 specimens with the highest and lowest HER, respectively. For the low-HER specimen, the grayscale distribution was wide, and two peaks were distant, resulting in a low peak count. In contrast, the high-HER specimen exhibited a concentrated grayscale distribution, resulting in concentrated peaks near grayscale value of 60. The grayscale difference and frequency difference between the top two peaks show different tendencies for each specimen. The differences in frequency and grayscale values between the two peaks were calculated and used as features.

## 4. HER Prediction Model

After extracting over hundreds of features, a Gaussian process regression (GPR) prediction model was built. GPR is a non-parametric supervised learning method, introduced in Section 4.1. Only several features were selected using GPR, details and their performances of which are discussed in Section 4.2. The selected features are analyzed phenomenologically and their relation with HER and punching process parameters are given in Section 4.3.

### 4.1. Gaussian Process Regression (GPR)

GPR is a supervised non-parametric learning method that does not assume any functional shape. As the structure of the model relies on a dataset, it has been used in fields in which the physical phenomena or mechanisms have not yet been explained, such as the prediction of additive manufacturing (AM) product quality [37,38], AM optimization [39], and AM modelling [40].

GPR model for predicting $\hat{y}(\hat{X})$ based on observation y(X), with independent and identically distributed Gaussian noise with variance $\sigma_{n}^{2}$, is given [41] as

(6) $$\hat{y}|X,y,\hat{X}\sim N\left(\mu \left(\hat{y}\right),\Sigma \left(\hat{y}\right)\right)$$

where the mean $\mu (\hat{y})$ is given by

(7) $$\left(\hat{y}\right)=H^{T}\overset{-}{\beta }+K^{T}\left(\hat{X},X\right){\left(K\left(X,X\right)+\sigma_{n}^{2}I\right)}^{-1}\left(y-H^{T}\overset{-}{\beta }\right)$$

where K is the kernel function, and H is the explicit basis. $\overset{-}{\beta }$ is vector of basis coefficients [41] given using

(8) $$\overset{-}{\beta }={\left(H\left(K\left(X,X\right)+\sigma_{n}^{2}I\right)H^{T}\right)}^{-1}H{\left(K\left(X,X\right)+\sigma_{n}^{2}I\right)}^{-1}y$$

In GPR, the covariance function, called the kernel function K, describes how close the input data of two observations $x_{i},x_{j}$ are. Most kernel function contains Euclidean distance terms. If the calculated kernel function is small, the predicted HER values $\hat{y}$ are close to each other. Because the underlying relationship between the features and the HER value is unknown, the hyperparameters, including the kernel function, are optimized within the predetermined iteration.

The optimized kernel function is isotropic rational quadratic given [41,42] using

(9) $$k\left(x_{i},x_{j}\right)=\sigma_{f}^{2}{\left(1+\frac{{\left\|x_{i}-x_{j}\right\|}^{2}}{2\alpha \sigma_{l}^{2}}\right)}^{-\alpha }$$

where $\sigma_{f},\sigma_{l},\alpha$ are hyperparameters. $\sigma_{f}$ is the standard deviation of the signal. The features are z-scored and $\sigma_{f}=0.7071$ for normalized features. $\sigma_{l}$ is characteristic length-scale and α is positive-valued hyperparameter, which were optimized.

### 4.2. Result of HER Prediction

The prediction performance of three models with different feature inputs were compared: (1) The first model used only the process parameters, that is, the material properties (Young’s modulus, yield strength, and ultimate tensile strength) and punching parameters (clearance, punch angle, and punch-edge radius); (2) The second model used features from only monitoring signals and edge images; (3) The third model used all the feature categories. The input features of the second and third models were optimized with sequential feature finding, a straightforward feature selection method that adds the feature sequentially to decrease the loss criterion. The loss criterion was set as the 50-fold cross-validation mean squared error (MSE) of GPR on a linear basis.

**Figure 13:**
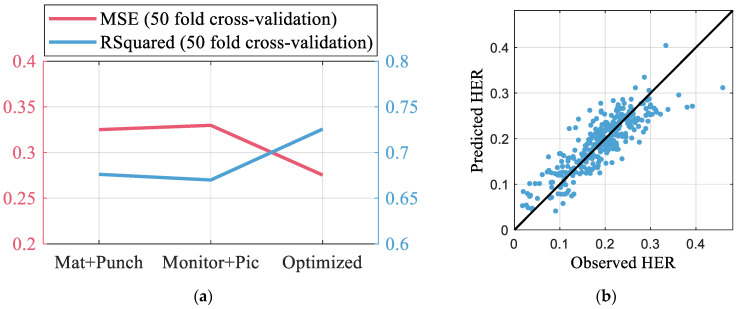
Result of HER prediction with selected feature set. (**a**) Comparison of prediction performance. (**b**) Prediction result with optimized feature set.

Figure 13a shows the MSE and $R^{2}$ values of the GPR models. The prediction with the process parameter alone, i.e., material properties and punching condition only, results in $R^{2}$ of 0.68, as the HER had a large variation within the same conditions. Without any information regarding the material and punching conditions, prediction using the monitoring signal and edge image can result in the same MSE. If the features are optimized, $R^{2}$ increases to 0.73, which is a 7.3% increase from the prediction using only the process parameter. The prediction results obtained using the optimized feature set are shown in Figure 13b.

### 4.3. Feature Analysis

**Figure 14:**
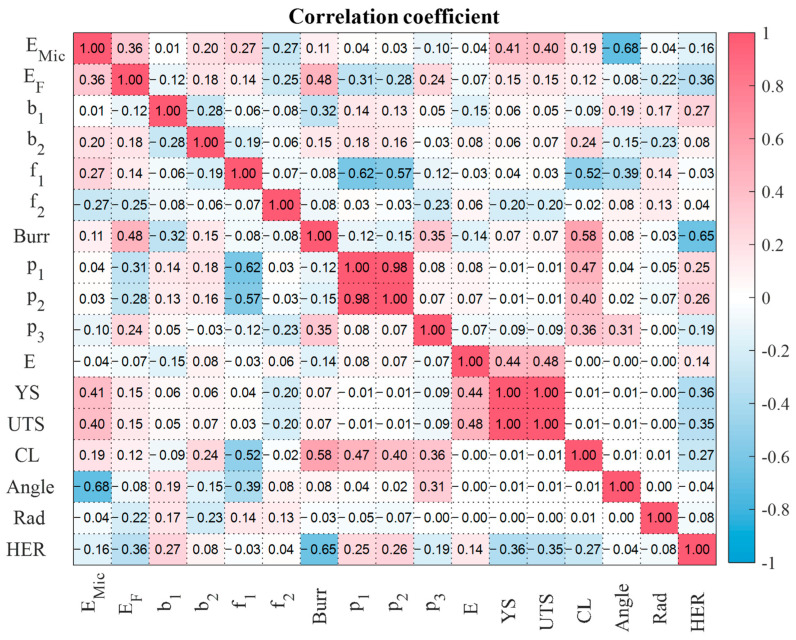
Correlation coefficient between optimized features and HER.

Table 4 presents the features of the prediction model. In addition to the sequentially optimized features, the material properties and punching parameters were included to analyze their effect. The selected monitoring signals were the sound and punch loads, with the selected feature only being the signal energy. The maximum signal value for the punch with angle may not adequately represent the characteristics of punching characteristics owing to the sequential pulse shape, as shown in Figure 8. Eight features of the edge panoramic images were selected. Among these, burr length was a widely known feature, whereas the other seven were newly suggested features. Figure 14 depicts the correlation coefficient between the sequentially selected features and HER. Some feature pairs, such as $p_{1}$, $p_{2}$ and YS, and UTS have similar characteristics and show a similar correlation coefficient, yet they were chosen in the sequential feature selection process.

Figure 14 shows that the edge radius had little effect on the HER, although a similar radius was found to have an effect on the Al-6000 series [25]. The effect of the edge radius was only apparent under limited conditions, such as a clearance of 24% and punch angle of 0°. In AHSS, the large variation in HER dominates the effect of the edge radius.

**Figure 15:**
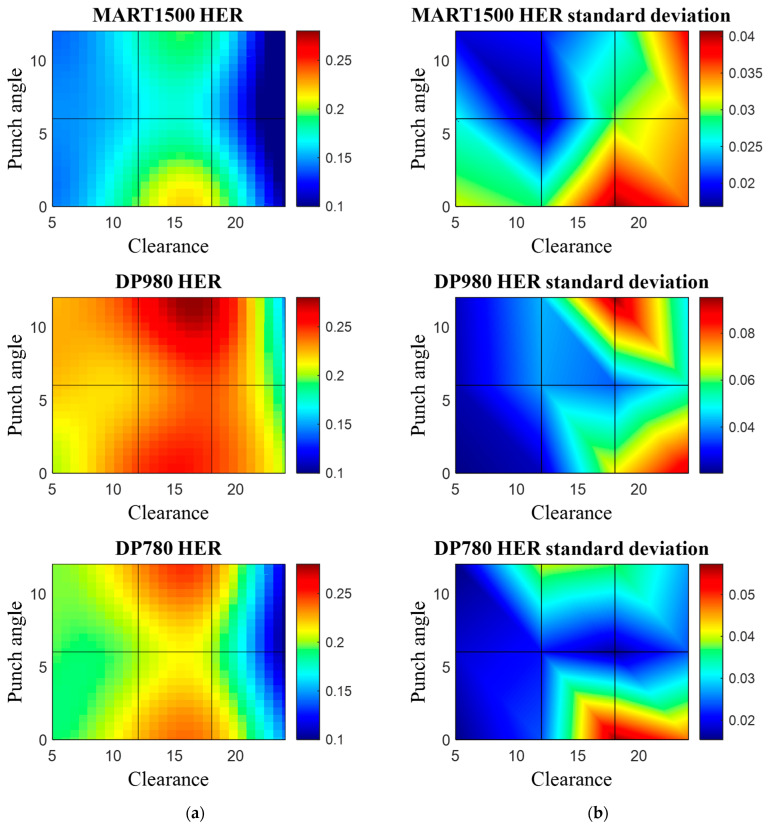
HET results with respect to punching parameter. (**a**) HER value with respect to material, clearance, and punch angle, fitted with GPR. (**b**) Standard deviation of HER with respect to each material, clearance, and punch angle.

Figure 15a shows the effect of clearance and punch angle on HER, and Figure 15b shows the standard deviation of the HER under the corresponding punching condition. HER values of the three AHSSs have the same tendency as the true uniform strain (Table 2). The punching condition with a high HER was always accompanied with a high variation of HER for all three materials, which implies the formation of an ill-structured phase even under the acceptable punching condition. All AHSS had a maximum HER approximately 18% clearance, but with a large variation. MART1500 exhibited the highest HER value of 18% 0° and the HER standard deviation was 0.04, which was 1/5 of the mean HER value.

**Figure 16:**
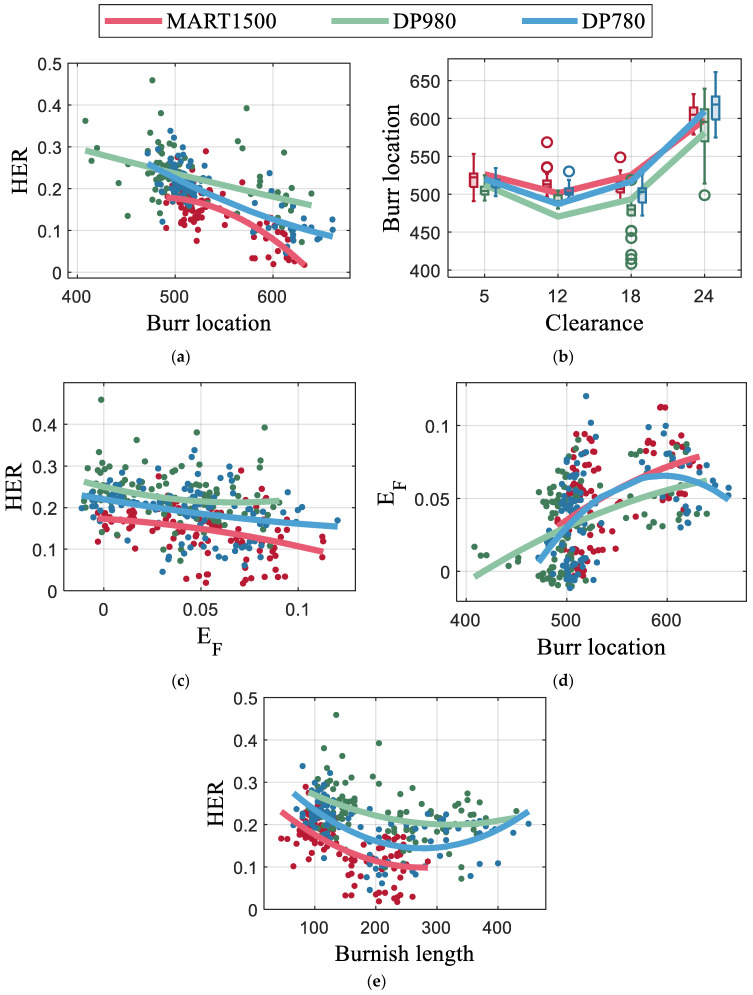
Effects of features: relation of burr to (**a**) HER and (**b**) clearance; relation of punch load to (**c**) HER and (**d**) burr; and relation of burnish length to (**e**) HER.

The burr location had the highest correlation coefficient of 0.6 to HER. The relation between the burr location and HER is plotted in Figure 16a using interpolation of the quadratic polynomial. The burr locations were not continuously distributed; instead, they were discretized. Figure 16b shows that the burr length increased rapidly at a clearance of 24% where the HER decreases rapidly for all AHSS materials.

It was observed that the HER increased with decreasing load energy $E_{F}$ (Figure 16c). The punching condition with lower energy consumption yielded a better edge quality. The correlation coefficient between $E_{F}$ and burr location was 0.5. A long burr is accompanied by a high load energy (Figure 16d). This indicates that the monitoring signal can be used to estimate the length of the burr, which is difficult to measure, whereas the monitoring can be completed in real-time. The energy of the sound signal $E_{Mic}$ had a relatively low correlation coefficient of −0.2 with HER, because $E_{Mic}$ was significantly affected by punch angle, and the effect of punch angle on HER is nonlinear (Figure 15). Additionally, $E_{Mic}$ is more sensitive to the material strength.

The burnish length is a conventionally used feature; however, it is not selected for sequential feature optimization. The relationship between burnish length and HER can be found on the Figure 16e. For all materials, the HER decreased as the burnish length increased, and the tendency weakened for extremely long burnish. A limitation of the burnish length is that the geometrical characteristics of the inhomogeneous edge formed by the asymmetric punch cannot be represented by the burnish length alone. The length of the fracture zone has an opposite tendency to that of the burnish length because most of the area of blanked edge was divided into burnish and fracture zone.

Seven features, related to the statistics of the panoramic image, were selected. $b_{1}$ and $b_{2}$ are features related to the grayscale distribution of the burnish zone, while $f_{1}$ and $f_{2}$ are that of the fracture zone. $p_{1},p_{2},$ and $p_{3}$ are the features related to the peaks of the grayscale histogram, as introduced in the Section 3.2.

**Figure 17:**
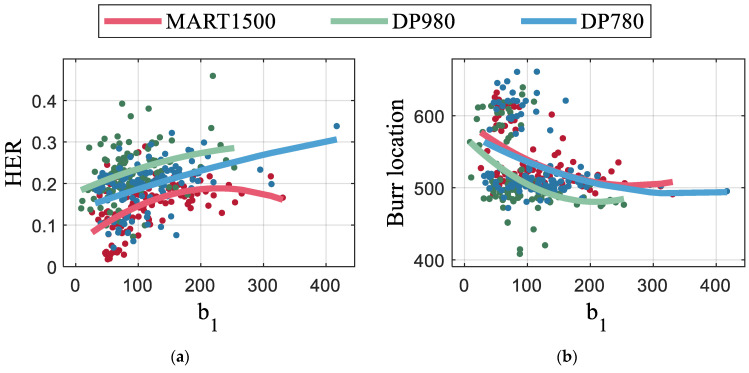

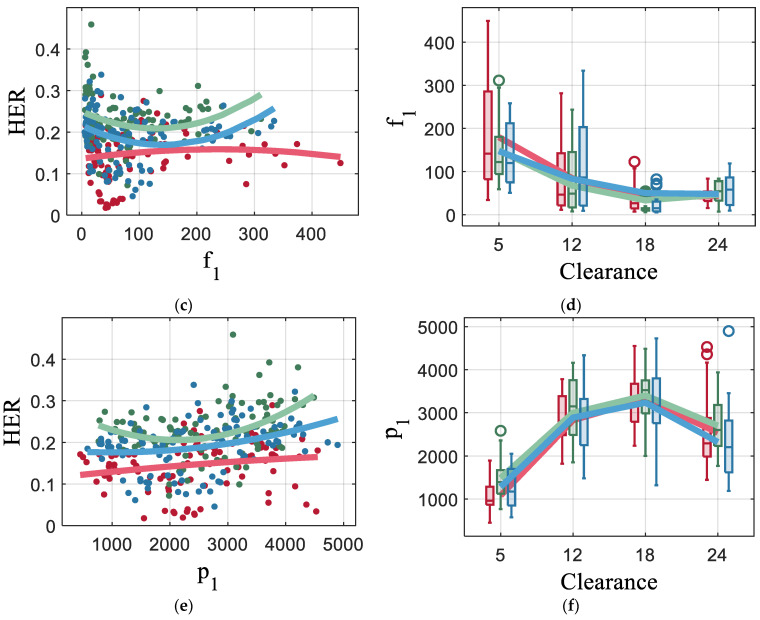
Effects of statical features of image: burnish-related features to (**a**) HER and (**b**) burr; fracture-related feature to (**c**) HER and (**d**) clearance; and peak-related feature to (**e**) HER and (**f**) clearance.

**Figure 18:**
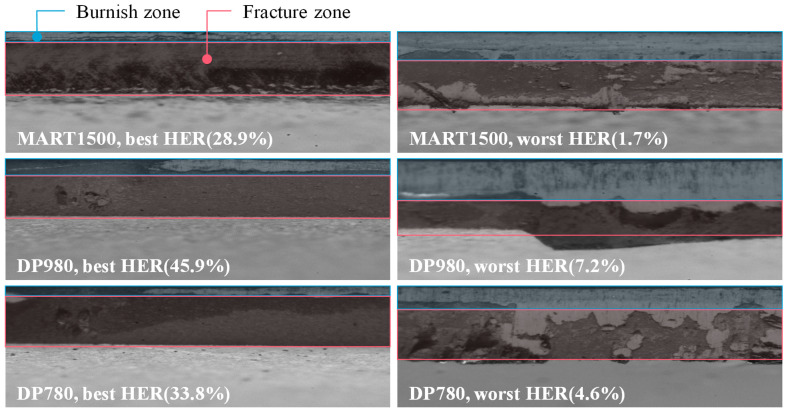
Edge image for all three materials with the highest HER and the lowest HER.

Burnish-related feature $b_{1}$ and $b_{2}$ are the grayscale deviations in the burnish zone. $b_{1}$ is the grayscale variance of the mean of the image column, and $b_{2}$ uses kurtosis instead of variance. The correlation coefficient for HER was higher for $b_{1}$ than for $b_{2}$. The relation between $b_{1}$ and HER is shown in Figure 17a. The grayscale variance in the burnish zone is related to short and discontinuous burnish. Figure 18 depicts the blanked edge surfaces of all the materials specimen with the highest and lowest HER. All the specimens with the highest HER exhibited extremely short burnish. A partially vanished burnish was observed for DP980 and DP780 specimens with a high HER, due to the anisotropy of punch with angle. On the other hand, the specimen with the lowest HER exhibited long burnish. Although the low-quality edges showed irregular and fluctuating burnish boundaries, the long burnish length reduced the variation in grayscale. In contrast, for short burnish, a relatively small fluctuation in the boundary has a significant effect on statistical features. Furthermore, Figure 17b shows a negative correlation between $b_{1}$ and the burr location, indicating that when the burnish is formed such that the geometry is favorable for the HER value, the burr length decreases.

Similar to the burnish zone, features of the fracture zone were also related to the grayscale variation. The selected features $f_{1}$ and $f_{2}$, which were variance and skewness of the grayscale, respectively, were affected by the length of the fracture and large fluctuations in the fracture boundaries, such as $b_{1}$ and $b_{2}$. In addition, the double burnish and uneven burr increased the grayscale variance. The variance of fracture zone had more influencing factors than the burnish zone, and the correlation between the HER and $f_{1}$ was more complicated, unlike the burnish zone. In Figure 17c, DP780 and DP980 show a similar nonlinear tendency, whereas MART1500 is insensitive to the HER. $f_{1}$ had a negative correlation with the clearance, as shown in Figure 17d. Because the clearance has a nonlinear relationship with the HER, as depicted in Figure 15, $f_{1}$ also has a nonlinear relationship with the HER. Feature $f_{2}$ was similar to $f_{1}$ but it was more sensitive to the material and insensitive to the punching parameters (Figure 14).

Lastly, three features related to the peaks of the fracture zone were selected and denoted as $p_{1},p_{2}$, and $p_{3}$. $p_{1}$ was the standard deviation of the difference between the frequencies of the grayscale values of the top two peaks at the fracture. The extraction of peaks of frequencies is described in Section 3.2 and Figure 12. $p_{2}$ is similar to $p_{1}$, but uses variance instead of the standard deviation. Therefore, $p_{1}$ and $p_{2}$ have similar correlation coefficients. $p_{1}$ and the HER have a positive correlation, as shown in Figure 17e. If a double burnish and uneven burr is formed in the fracture zone, the grayscale frequency of the peak is relatively low (Figure 12), and the difference between the frequencies of the two peaks is small and uniform. Because the double burnish and uneven burr induces a low HER, a positive correlation between $p_{1}$ and the HER was formed. It was found that $p_{1}$ had a correlation coefficient of 0.5 with clearance, and the maximum of $p_{1}$ can be found around a clearance of 18% (Figure 17f) where the HER is high for all the materials (Figure 15a).

$p_{1}$ was related to the difference between the peak frequencies, whereas $p_{3}$ was related to the difference between the grayscale values of the peaks. The specimen with a high HER exhibited a single uniform peak location (Figure 12). In contrast, the specimen with a low HER exhibited double or multiple peak locations. Owing to this phenomenon, the difference between the grayscale value was high for the specimen with a low HER, increasing $p_{3}$. Hence, $p_{3}$ was negatively correlated with HER. $p_{3}$ had a positive correlation coefficient of 0.3 with the punch angle, which indicated that double burnish was affected by the punch angle.

## 5. Discussion

The suggested features were based on the geometries of the entire edge and have been shown to be related to punching parameters and HER. When using only monitoring signals and process parameters, the prediction performance was similar to that based solely on punching parameters. This suggests that the estimation of HER is possible using the suggested features, even when changes in the punching process are unknown, such as wear. Although the features have a wide distribution, similar tendencies towards process parameters and HER were observed for the three materials studied. For the extremely high-strength material MART1500, the correlation was weaker in the variation of fracture zone.

Some features, such as the variation of the fracture zone, showed a high correlation with punching parameters, allowing for prediction to the HER without the punching parameters. Other features, such as burnish variation, had low correlation with punching parameters but higher correlation with burr location and HER. Improved prediction performance with these features indicates that the suggested features, which are macroscopic observations of edge quality, correct for variations in HER that are not captured by deterministic process parameters. Feature $f_{2}$ had a low correlation with both HER and punching parameters, indicating a nonlinear relationship with the HER.

While the variation in HER can be corrected through monitoring and measurement, the HET process also introduces deviations in HER. There are no automatic quantitative criteria available to assess HET in AHSS. Previous studies have shown that failure tests of AHSS with load drop criterion, such as three-point bending, are not feasible because of the load drop delay after cracking [43,44].

## 6. Conclusions

In this study, a prediction of HER was made with data with three categories: (1) pre-punching data which consist of tensile properties of AHSS and punching parameters including clearance, punch angle, and punch-edge radius; (2) punch monitoring data of sound, vibration, and force; (3) post-punching measurement where the data were the entire edge image. The image analysis combined with the process monitoring was applied to provide macroscopic, rapid, and non-destructive evaluation of the edge quality. Statistical features of the panoramic image and the intensity of the signal had been used for prediction. The sequentially optimized features were those related to an inhomogeneous surface, such as the grayscale variance. Based on the experiment, the following conclusions were drawn for DP780, DP980, and MART1500.

1. For selected AHSS, the clearance and punch angle have distinct effects on the HER, whereas the effect of the punch-edge radius was limited to certain conditions of the clearance and punch angle.
2. The burr location had the highest correlation with the HER and was heavily affected by clearance. Specifically, the burr length rapidly increased when the clearance increased to 24%.
3. For all materials, HER decreased as the load energy used in the increased punching process. A long burr is accompanied by high punching energy.
4. The variation in the grayscale in the burnish and fracture zones affects the HER value. As the grayscale variation at the burnish zone increases, the HER increased, and the burr length decreased. If a double burnish and uneven burr appears in the fracture zone, grayscale variation is increased while HER is reduced. The variation in the fracture zone was significantly affected by the clearance and punch angle.
5. The prediction model that used only the monitoring signal and image exhibited the same performance as the model that used only the material properties and punching parameters. This can be applied to situations where a change in the punching process is unknown, such as wear. Prediction performance increases by 7.3% when all types of features were used with feature optimization.

## Figures and Tables

**Table 1:** List of input data, data measurement method, and feature type.

Data Category	Data Type	Measurement Method	Feature Type
Pre-punching	Material property	Tensile test	Raw value of E, YS, UTS
	Punching process parameter	-	Raw value of clearance, punch angle, punch-edge radius
Punching monitoring	Sound	Microphone GRAS 146AE (Max.f 20 kHz, Max.dB 133 dB)	Statistical (Max, integral)
	Acceleration	Accelerometer Kistler 8763B050BB (Max.f 7 kHz, Max.acc 50 g)	
	Force	Load cell (Max.f 20 Hz)	
Post-punching	Edge image	Charge-coupled device (CCD) camera DMK 33GP031 (Resolution 5 MP, Pixel 2.2 μm)	Statistical (Mean, var, std, kurt, skew, …)
Edge quality	Hole expansion ratio	Hole expansion test (ISO 16630)	Raw value

**Table 2:** Result of tensile test for DP780, DP980, and MART1500.

Material Properties	DP780			DP980			MART1500		
	RD	45°	TD	RD	45°	TD	RD	45°	TD
Yield strength (YS), MPa	1003	984	1036	1215	1210	1235	1646	1618	1660
Ultimate tensile strength (UTS), MPa	814	814	777	952	964	1053	1465	1417	1420
Young’s modulus (E), MPa	216,493	19,744	191,775	229,394	219,254	222,316	214,697	217,889	215,092
True uniform strain, %	9.24	9.02	9.13	11.03	10.43	11.1	4.9	4.02	4.04

**Table 3:** Experimental conditions for punching.

Parameter	Value
Materials	DP780, DP980, MART1500
Clearance	5, 12, 18, 24%
Punch angle	0, 6, 12°,
Punch-edge radius	50, 125, 250 μm
Punch speed	5 mm/s
Repetition	3
Total specimen	324

**Table 4:** List of optimized features.

Data Category	Data Type	Feature	Symbol
Pre- punching	Material property	Young’s modulus, yield strength, and ultimate tensile strength	E YS UTS
	Punching parameter	Clearance, punch angle, and punch edge radius	CL Angle Rad
Punching monitoring	Sound	Signal energy	$E_{Mic}$
	Force	Signal energy	$E_{F}$
Post- punching	Edge image	Variance of grayscale mean at burnish	$b_{1}$
		Kurtosis of grayscale mean at burnish	$b_{2}$
		Variance of grayscale mean at fracture	$f_{1}$
		Skewness of grayscale mean at fracture	$f_{2}$
		Burr location	Burr
		Standard deviation of the difference between the frequencies of the grayscale value of top two peaks at fracture	$p_{1}$
		Variance of the difference between the frequencies of the grayscale value of top two peaks at fracture	$p_{2}$
		Variance of the difference between the grayscale values of top two peaks at fracture	$p_{3}$

## Data Availability

Data are available with the permission of all authors.
